# The Use of Prebiotics from Pregnancy and Its Complications: Health for Mother and Offspring—A Narrative Review

**DOI:** 10.3390/foods12061148

**Published:** 2023-03-08

**Authors:** Cielo García-Montero, Oscar Fraile-Martinez, Sonia Rodriguez-Martín, Jose V. Saz, Rocio Aracil Rodriguez, Juan Manuel Pina Moreno, Javier Ruiz Labarta, Natalio García-Honduvilla, Melchor Alvarez-Mon, Coral Bravo, Juan A. De Leon-Luis, Miguel A. Ortega

**Affiliations:** 1 Department of Medicine and Medical Specialities, Faculty of Medicine and Health Sciences, University of Alcalá, 28801 Alcalá de Henares, Spain; 2 Ramón y Cajal Institute of Sanitary Research (IRYCIS), 28034 Madrid, Spain; 3 Service of Pediatric, Hospital Universitario Principe de Asturias, 28801 Alcalá de Henares, Spain; 4 Department of Biomedicine and Biotechnology, Faculty of Medicine and Health Sciences, University of Alcalá, 28801 Alcalá de Henares, Spain; 5 Department of Public and Maternal and Child Health, School of Medicine, Complutense University of Madrid, 28040 Madrid, Spain; 6 Department of Obstetrics and Gynecology, University Hospital Gregorio Marañón, 28009 Madrid, Spain; 7 Health Research Institute Gregorio Marañón, 28009 Madrid, Spain; 8 Immune System Diseases-Rheumatology and Internal Medicine Service, University Hospital Príncipe de Asturias, CIBEREHD, 28806 Alcalá de Henares, Spain

**Keywords:** prebiotics, pregnancy, newborn, gut microbiota, lactation

## Abstract

Pregnancy involves a metabolic reprogramming that includes changes in the gut microbiota composition in women. Evidence shows that maternal dysbiosis is linked to neonatal dysbiosis, and this factor can determine health status in adulthood. Although there is little literature available on this topic, high heterogeneity is a limitation when examining nutritional interventions. Information has been gathered to contrast the benefits of prebiotic usage, specifically in pregnancy, in its possible complications and in newborns’ gut microbiota development. The objective pursued in this brief narrative review is to provide a clear summary of relevant content when searching with regard to the use of prebiotics in pregnancy, the effects in prenatal and postnatal periods, and to help in clinical decision-making in pregnancy management and lactation. A search has found that the nutritional status of the pregnant mother is key for the earliest microbial colonization in newborns, and thus intervention programs from pregnancy could assure better outcomes in both the mother and offspring. In this sense, prebiotics (administered to mothers who breastfeed or provided in formula milk) are feasible and cost-effective elements that can prevent allergies, colic, and other maladies in newborns.

## 1. Introduction

Pregnancy entails a complex period in a woman’s life, with many associated metabolic changes and possible complications. In recent years, multiple advances have been made in a short period of time with regard to the management of healthy and pathological gestation towards a better quality of life during this period. The gut microbiota is highly altered in this period. Pregnancy is also associated to shifts in nutritional requirements, the endocrine system, and the gut microbiota. Therefore, nutritional interventions are a feasible and cost-effective adjuvant way to address those changes and achieve better outcomes [1,2]. In this sense, vitamins are commonly recommended by physicians, but other bioactive compounds are overlooked. Moreover, nutritional programs that include a nutritionist do not normally accompany pregnancies; however, some women often resort to strict diets in the postpartum stage [3,4].

It is important to provide professionals with different adjuvant strategies when giving nutritional advice to a pregnant woman beyond the usual supplements and promoting a healthy diet pattern from the beginning of this stage of a woman’s life [5]. To acquire better outcomes in the quality of life of the mother and her offspring, it is important to highlight the use of prebiotics as promising modulators of the intestinal microbiota. These strategies may also be useful to prevent or combat certain pregnancy-associated complications such as gestational diabetes, preeclampsia, or infections, among others, and to prevent future maladies such as asthma or allergies in children [6,7].

Probiotics are living microorganisms that can be consumed in certain foods like yogurt, kefir, kombucha, tempeh, and miso soup, or taken as supplements. These products normally harbor beneficial bacteria such as *Lactobacillus* spp., *Bifidobacterium* spp., *Streptococcus* spp. and others, and also yeasts, including *Saccharomyces* spp. All of these genera can transiently colonize the intestinal mucosa and aid with the host’s flora metabolism. However, less is known about the combination of these bioactive compounds with their favorite substrates, designed as “prebiotics”. They are dietary components with complex carbohydrates that are selectively utilized by host microorganisms rather than human cells, and confer a health benefit according to the International Scientific Association of Prebiotics and Probiotics (ISAPP) [8,9].

Limited evidence and high heterogeneity may prevent the consideration of the use of prebiotics in pregnancy. Thus, the objective of the present review is to describe the available evidence from the last 10 years related to the use of prebiotics and to develop some guidelines that can be useful for the clinical management of the health of pregnant women.

## 2. What Are Prebiotics and What Types Are There?

The term “prebiotics” was first coined by professors Emeritus Marcel Roberfroid and Glenn Gibson in a 1995 publication, where they explained the selective growth of colonic bifidobacterials boosted by the intact fibrous oligosaccharide inulin. They explained prebiotics as “nondigestible food ingredients that beneficially affect the host by selectively stimulating the growth and/or activity of one or a limited number of bacterial species already resident in the colon, and thus attempt to improve host health” [10].

Unusable by human cell enterocytes, prebiotics are therefore microbe food, usually consisting of plant dietary fibers with complex carbohydrates (short and long chain β-fructans [fructooligosaccharides, FOS and inulin], lactulose, and galactooligosaccharides, GOS) which can help the host flora, promoting their growth and/or boost supplemented probiotic effects. It is worthy of note that prebiotics can limit the growth of potential pathogenic bacteria as well. In 2008, the definition was refined to “dietary prebiotics”, which were described as “a selectively fermented ingredient that results in specific changes in the composition and/or activity of the gastrointestinal microbiota, thus conferring benefit(s) upon host health” [11].

This more recent definition is due to the nature of these bioactive components, not only being carbohydrates but also some polyphenols such as flavonols. The classification of prebiotics involves five groups: fructans (FOS and inulin), GOS, resistant starch and glucose-derived oligosaccharides (e.g., polydextrose), pectin-derived oligosaccharides, and non-carbohydrate oligosaccharides entailing polyphenols (e.g., cocoa flavonols). All of these primarily stimulate Lactobacilli and Bifidobacteria, as well as Enterobacteria, Bacteroidetes and Firmicutes [12].

Furthermore, currently the term “synbiotic” is commonly used as well, referring to dietary supplements that contain both pre- and probiotics. In 2019, the ISAPP updated its definition to “a mixture comprising live microorganisms and substrate(s) selectively utilized by host microorganisms that confers a health benefit on the host” [13].

## 3. Prebiotics for Healthy Pregnant Women

In a 2018 meta-analysis, little evidence was collected about the efficacy of prebiotics, but in combination with probiotics it was concluded that the risk of preterm birth was not increased or decreased due to the intake of these compounds during pregnancy [14]. Despite this argument, other objectives can be pursued to contrast the benefits of prebiotics in pregnancy compared to not using them.

A primary reason to consider the recommendation to take prebiotics in pregnancy lies in the changes in the nutritional requirements of a pregnant woman. Sheridan et al. explained that gut microbiota is highly altered in this period of a woman’s life, between the first and third trimesters, with an increase in the relative abundances of Proteobacteria and Actinobacteria, along with a decrease of bacterial diversity in general. With a view to promoting a healthy microbiota in this population, they then proposed that these microbial alterations should be addressed by designing more robust human studies with pro- and prebiotics applications [15].

In fact, pregnancy has been categorized by some authors as a “stressor” that reduces barrier function due to elevated serological markers of elevated intestinal permeability (as with zonulin or lipopolysaccharide), and they argue that this should be faced by restoring barrier function with supplements including prebiotics [16]. However, it should be mentioned that microbial changes during pregnancy can also be part of the immunosuppression that occurs in maternal utero to allow paternal antigens and to prevent the rejection of the embryo. To achieve this, the mother should have increased IgA and phagocytic cell concentrations and diminished vaginal pH thanks to *Lactobacilli*. These favorable changes are in relation to preventing vertical infections at the moment of birth [17]. Thus, it seems important to protect the vaginal microbiota.

In a randomized double-blind placebo-controlled trial with maternal intake FOS programming during the third trimester, there was a significant increase in fecal *Bifidobacterium* spp. and *Bifidobacterium longum* in the intervention group at the end of pregnancy [18]. Another randomized controlled trial in Indonesian pregnant women evaluated the administration of milk fortified with prebiotic, probiotic, DHA and micronutrients, observing notable increases in the fecal concentration of the organism used as the probiotic [19]. Therefore, this evidence indicates that probiotic colonization is successful when combined with prebiotics and other micronutrients.

Another randomized controlled trial assessed the effectiveness of synbiotic food in pregnant women for 9 weeks and its impact on insulin levels. Synbiotics containing *Lactobacillus sporogenes* and inulin showed significant differences with lower serum insulin levels in the intervention group versus the control group [20].

A meta-analysis regarding the safety of pro- and prebiotics use in pregnancy and lactation found that adverse effects were rare and normally related to changes in stool consistency and did not pose risks to the mother’s or child’s health [21]. It is also true that more scientific evidence is needed before drawing any relevant conclusions in the administration of prebiotics in healthy pregnant women. However, this preliminary evidence seems to be encouraging regarding the potential use of prebiotic supplementation in pregnancy. Nevertheless, it would be fruitful to study which groups of women would benefit the most and how to optimize prebiotic formulas and synbiotics in more detail.

**Figure 1:**
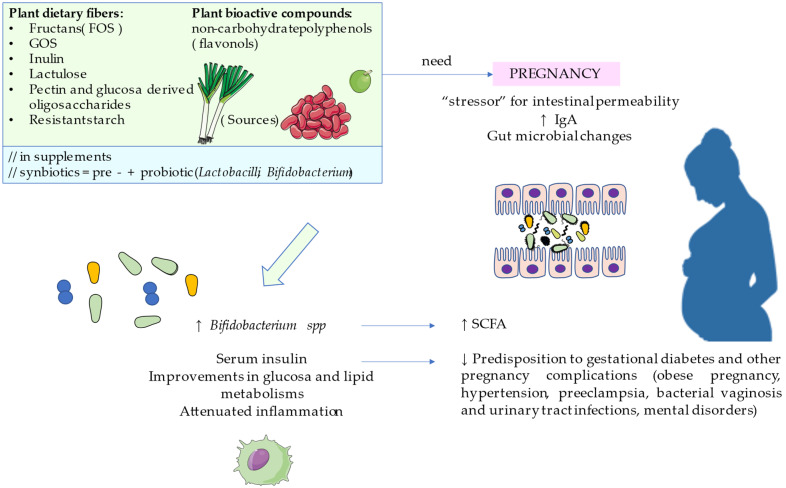
The effect of prebiotics on pregnancy.

Some highlights can be summarized in Figure 1.

## 4. Prebiotics for Pregnancy Complications

Many pregnancies face adversity with hypertension and metabolic problems such as gestational diabetes mellitus, obese pregnancy, or metabolic syndrome. All of these complications may have an impact on maternal health on the mother’s and also on newborn’s health. In fact, these complications have been related to infant allergy, asthma or skin problems, as will be discussed later.

Many studies are in agreement that pro-, prebiotics, and synbiotics have health benefits in terms of preventing adverse pregnancy outcomes. In this line, Sohn and Underwood reviewed that dysbiosis is a key factor involved in increasing the risk of pre-eclampsia, diabetes, infection, preterm labor, and (later) childhood atopy. It has been demonstrated that this maternal dysbiosis goes hand in hand with neonatal dysbiosis, which causes colic in infants. Therefore, the administration of pro- and prebiotics during pregnancy and lactation was recommended as a safe option to optimize these periods of life and prevent adverse outcomes [22]. Next, we will comment on some of the complications associated with pregnancy for which some results have been found according to the search criteria. Most of them are mere proposals that still lack clinical rigor but represent new horizons in the management of these maladies.

### 4.1. Obese Pregnancy

Obesity in pregnancy entails a maternal body max index ≥30. Obese women may suffer from dysbiosis and metabolic impairment, which can cause damage to the fetus or even a miscarriage. This condition can increase the risk of other pregnancy complications such as gestational diabetes mellitus, hypertension, preeclampsia, venous disease, or even venous thromboembolism. The consequences for the fetus can be premature birth, neonatal death, and the increased risk of metabolic disorders in later life.

Wiedmer and Herter-Aeberli narrated the intergenerational cycle of obesity: the mother’s dysbiosis entails shifts in maternal metabolites and the placental microbiome, having implications in fetal programming. The risk of obesity for the child is very high. For this issue, there have been more animal models examining prebiotics use than human studies, which normally only evaluate probiotics. Prebiotic supplementation in preclinical models has demonstrated a clear decreased risk of obesity in the offspring, but more evidence is required [23]. Zhou and Ziao summarized the mice and rat models of maternal prebiotic exposure to GOS, inulin, FOS, oligofructose, caprine milk oligosaccharides, and some derivatives of all of these. The effects observed on offspring included reduced symptoms of allergic asthma, the prevention of food allergies, a lessening in the severity of atopic dermatitis, less fat mass, better immune response, and reduced obesity risk [24].

Prebiotic use has yet to be explored with regard to reducing the obesity risk in children from obese pregnancies, but they may cause an amelioration of maternal metabolic health by the production of probiotic short chain fatty acids. Other authors agree that the future research ought to be focused on intervention strategies combining B vitamin deficiencies and targeting gut dysbiosis in obese mothers through pro- and prebiotics [25].

### 4.2. Gestational Diabetes

Women with GDM exhibit pancreatic β-cell dysfunction on a background of chronic insulin resistance during pregnancy, along with changes in other systems and organs [26]. This problem normally appears only during pregnancy but can also have metabolic consequences for the offspring. The management of multifactorial disease gestational diabetes mellitus (GDM) is also contemplated from the perspective of dysbiosis. Some authors propose soy oligosaccharides as intestinal modulators involved in the alleviation of insulin resistance and other related pathological events such as oxidative stress in women with GDM [27]. They promote the advantages of certain foods such as soy, which has prebiotics, but also has polyphenols that can form a synergy to enhance its effects [28]. Similarly, the use of synbiotics have also shown notable benefits on the clinical management of GDM. According to a meta-analysis conducted by Zhou et al. [29], this type of intervention has demonstrated significant improvements on glucose and lipid metabolism, as well as having an anti-inflammatory and antioxidant effect on diet controlled GDM patients, reducing the risk of fetal hyperbilirubinemia, fetal macrosomia, and limiting newborn weight. Similarly, a systematic review and meta-analysis also demonstrated that the use of synbiotics ameliorated insulin resistance in GDM women [30]. However, a recent meta-analysis undertaken by Łagowska et al. [31] found that similar benefits were observed when either probiotics or synbiotics were used, this probably resulting from the fact that not all Lactobacillus and Bifidobacterium strains have the ability to ferment certain prebiotics used. Thus, more efficient synbiotic combinations arises as a promising and potential field of research to evaluate in pregnant women, and particularly in GDM patients.

### 4.3. Hypertension and Preeclampsia

Preeclampsia is a kind of complication characterized by high blood pressure and proteinuria, leading to kidney and multisystem damage. It also entails a danger to the fetus. Gynecological studies regarding a well-functioning vaginal microbiome indicate a benefit of this depending on the gut microbiome. Pro- and prebiotic use stimulate gut-derived metabolites such as butyrate that attenuate inflammation [32]. Other authors have also mentioned the potentiality of pro- and prebiotics in tandem in a context of nutritional interventions to manipulate oral microbiota as well. They found that this microbial ecosystem is also a key to cardiovascular health during gestation, and therefore may aid in the treatment and prevention of hypertensive pregnancy disorders including hypertension and preeclampsia [33]. Conversely, in a recent systematic review and meta-analysis, Movaghar et al. [34] did not find any notable benefits from probiotics or synbiotics administration in women with hypertensive disorders and GDM, although due to the limited number of studies available, further efforts are required.

### 4.4. Bacterial Vaginosis

Other impairments in vaginal microbiota can provoke the occurrence of chlamydia. This imbalance can be present during pregnancy and cause premature labor or spontaneous miscarriage. This is another reason why an increasing number of scientists and doctors are contemplating the idea of nutritional strategies that help prevent this damage. Synbiotics consumption seems to effectively prevent recurrent urinary tract infections in women [35]. In the case of bacterial vaginosis, they suggest that a combination of probiotics and prebiotics should be applied instead of using antibiotics, which is risky for a pregnant woman. Systematic reviews found that pre/probiotic regimens have even higher cure rates than antibiotics [36]. This would also provide greater protection to the mother and the fetus.

### 4.5. Perinatal Mental Health

Currently, there are clinical trials exploring the role of gut microbiota modulators on mental health (i.e., Ecologic Barrier© -NCT04951687-) in the general population, suggesting a complex but an evident relationship between the gut and the brain. It is necessary that mental health in postpartum women be addressed. Perinatal mood disorders are common disabling conditions that can be treated through the so-called gut-brain axis. According to systematic reviews, promising limited evidence of lower incidence of anxiety and depressive symptoms in the perinatal period has been reported when supplementing with pro-, pre-, and synbiotics during pregnancy [37,38].

## 5. Prevention of Pediatric Disorders

### 5.1. Prebiotics in the Window of Opportunity

It is said that the first 1000 days of a human life are critical to determining health status, even in adulthood, so this period can be considered as a window of opportunity [39]. A review conducted by Firmansyah et al. [40] showed that β-(2,1)-linked linear fructans and inulin seems to be a potential prebiotic with a bifidogenic effect, causing the production of short chain fatty acids. Other effects found in newborns included a reinforced immunity against infections. These authors, again, defended prebiotics intake from pregnancy as part of good nutrition during this period, influencing neonates’ gut microbiota development [40]. In a randomized controlled trial mentioned above, there were significant increases in fecal *Bifidobacterium* spp. when supplementing mothers with FOS, but no difference was observed in neonates aged 1 month [18]. This result should only fit with the idea that longer term studies need to be accomplished now that first droppings and an incipient digestive system represent difficult elements for the measuring of fecal microbiota. In another systematic review, prebiotic supplementation during pregnancy and lactation did not show significant alterations in infant gut bacterial diversity or abundances, but the authors emphasized the heterogeneity in the study designs as well. In the same analysis, it was remarkable that probiotic supplementation during pregnancy and lactation did show the probiotic colonization of both infant gut microbiota and breastmilk microbiota [41].

It seems that most authors agree that there are certain favorable elements for establishing a healthy microbiome from birth: a vaginal mode of delivery, breastfeeding, and the mother’s good nutritional status. A damaging factor would be undernourishment in both the mother and the newborn. Finally, some factors would make an infant start with a certain disadvantage, such as formula milk feeding and caesarean delivery [2,40,41,42]. Some articles found under the search criteria for this narrative review were specifically related to the prebiotic composition of breastfeeding. So-called human milk oligosaccharides (HMO) provide a source of natural prebiotics provided by the mother, and will shape the gut microbial composition in newborn, diminishing the predisposition to allergy and autoimmune diseases in later life [42].

Interestingly, some reviews based on short-term studies have observed reduced daily crying time when administering probiotics compared to placebo. The same studies defend the application of oligosaccharide prebiotics to promote the growth of beneficial bacteria as treatments for allergy or intolerance, and for crying for babies with colic [43].

A premise from the ecological theory says that microbial community development is moved by priority effects that determine species arrival and their behavior, which can be favorably modulated by pro- and prebiotics, so therapies targeting gut microbiota are a potential choice [44].

In this context, the industry has been testing different formulas of milk during recent years, with a wide variety of dietary fibers and probiotics and prebiotics, including HMO, FOS, GOS, inulin and post-biotics (milk fermentation products) being used to modulate the gut microbiota sooner [45]. The common observation is an increase in bifidogenic bacteria and a decrease in opportunistic pathogens, in addition to a reduction of fecal pH, an increase in alpha diversity, and optimized calcium absorption [46]. Mimicking human milk is a challenge, nevertheless the rising evidence about breastfeeding’s composition in prebiotics that stimulates *Bifidobacteria* and *Lactobacilli* is worthy of note [47]. However, when breast milk is not available or adequate, HMO fortified formulas seem to be the best option [48].

In any case, that early colonization will determine gut microbiota composition after weaning. A randomized controlled trial studied the effect of a specific prebiotic mixture administered from the day of birth on bifidobacterial and lactobacilli counts. Examination was performed at 3, 6 and 12 months of age, finding that the supplemented group had more fecal bifidobacterial and lactobacilli compared to placebo, these differences being maintained six months later without further supplementation [49]. This type of explorative study shows that prebiotics, even in first days of life, are valuable in establishing a competent gut immune system.

**Figure 2:**
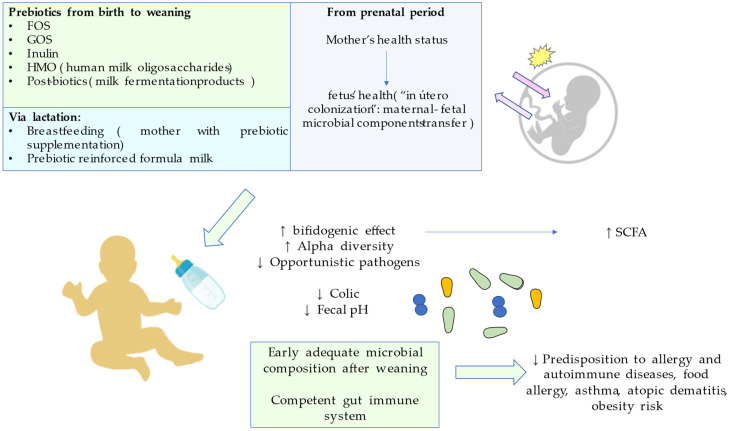
Prebiotics effects on fetus and newborn, from prenatal and lactation until weaning moment.

It is of note that, as discussed in a previous section, the health status of the mother during pregnancy is key for the neonate’s healthy microbiota. Del Toca et al. found that there are certain factors concerning the pregnant mother that will affect the early massive microbial colonization which starts at birth. These factors will shape the microbial composition, especially in the third trimester: diet, body mass index, weight gain during pregnancy, stress, and antibiotic or other drugs. Unhealthy patterns will also promote inflammation with accompanying bacterial translocation that will affect vertical transmission from the mother to the newborn. Moreover, they pointed that, although information related to fetal microbiota and its possible implication in newborn’s intestinal colonization is disputable, there are similarities in the placental microbiota, the amniotic fluid, and the meconium that propose a maternal-fetal microbial transfer [50]. These premises converge in the so-called “in utero colonization hypothesis”. Microbial DNA and cell rests from intestinal bacteria have been detected in those structures from term pregnancies without signs of unhealthy inflammation [51]. Despite unanswered hypotheses of prenatal origin in incipient microbiota, it is undeniable that a mother’s health during the entire pregnancy is key for a proper microbial colonization in the child. These highlights are summarized in Figure 2.

### 5.2. Caesarean Delivery

Furthermore, children born by caesarean section are at a disadvantage compared to children born vaginally. the bacterial charge is different, and this leads to a different immune system development process [52]. Specifically, babies delivered by caesarean section do not receive certain maternal *Bacteroides* stains from the vagina, and instead they receive a higher colonization of opportunistic pathogens such as the *Enterococcus*, *Enterobacter* and *Klebsiella* species, which are associated with the hospital environment. Furthermore, these infants have a similar composition to maternal skin and hospital setting with *Staphylococcus*, *Streptococcus* and *Clostridium*. Babies born vaginally have higher rates of *Bifidobacteria* and *Lactobacillus* in the first days of colonization and contain more microbial diversity in the following weeks. This different colonization is frequently associated with an increased risk of developing immune and metabolic disorders in adulthood [53,54,55].

For these reasons, many authors insist on targeting the gut microbiota during pregnancy and lactation with immunobiotics (both pro- and prebiotics), but there is not enough empirical data from clinical trials for caesarean infants. On one hand, it is true that stool samples from supplemented babies are softer and seem to be related to a lower level of pathogens. On the other hand, they reveal that the duration of supplementation to ensure a lasting beneficial effect is yet unknown and is another gap to fill in clinical studies [56]. Lista et al. agree with the idea of maternal and neonatal supplementation with functional nutrients to correct dysbiosis and reduce newborn disease [57]. Moya-Pérez et al. highlight the importance of these interventions for host immunity, metabolism, and the gut-brain axis to prevent neurodevelopmental disorders in caesarean children [58]. A recent systematic review noted that the sooner the intervention during pregnancy and lactation after cesarean delivery, the better the effects that are achieved, especially *Bifidobacterium* colonization. The results were even more favorable in breastfed infants from supplemented mothers. The utilized formulas included GOS, FOS, or bovine milk-derived oligosaccharides combined with probiotics from genera *Lactobacillus*, *Bifidobacterium*, *Propionibacterium*, and *Streptococcus* [59].

### 5.3. Preterm Labor

Preterm labor can be a consequence of previous pregnancy complications. Buffet-Bataillon et al. hold that by targeting maternal gut microbiota, breast milk would reinforcement the neurodevelopment in preterm infants. They believe that this manner could be much more powerful than fortified formula. As gut microbiota and central nervous system developments go hand in hand, targeting the maternal microbiota would stimulate not only the prebiotic effects of breastfeeding, but also its microbial composition. These new horizons would allow for the colonization and evolution of the preterm intestine [60].

### 5.4. Asthma and Allergy

Allergies and asthma represent two conditions that frequently occur together. Recent findings reveal that the gut microbiome is a crucial mediator of the pathogenesis of allergy and asthma. Environmental exposure during pregnancy can directly modulate the maternal gut microbiome and, subsequently, the infant’s risk of allergic disease and asthma.

The use of prebiotics in pregnancy has been evaluated to prevent allergies and asthma, and despite some mixed evidence obtained, the World Allergy Organization review found a lack of evidence to support the use of prebiotics during pregnancy according to current evidence [61,62]. Conversely, in a narrative review, Trambusti et al. collected different studies, evaluating the potential use of prebiotics for the prevention of pediatric asthma in prebiotic-rich formula milk [63]. The use of a formula milk containing a mixture of prebiotic oligosaccharides was associated with lower incidence for recurrent wheezing in 132 infants at risk of atopy, although the supplementation of non-human neutral and acidic oligosaccharides during the neonatal period did not reduce the incidence of allergies, bronchial hyper-reactivity, and respiratory infections in 113 preterm infants. Forsberg et al. summarized the main findings in the use of prebiotics in the primary prevention of asthma and allergies [64]. They found a meta-analysis of two studies with 249 infants reporting a reduction in infant asthma or recurrent wheeze in prebiotic-treated infants, and a single study reporting a significant reduction of the risk of developing a food allergy by the use of prebiotics. According to some authors, one of the most promising benefits from the use of prebiotics in the prevention of allergic diseases and asthma is their ability to induce the production of short-chain fatty acids, especially dietary fiber and oligosaccharides, leading to the activation of T regulatory cells and tolerance mechanisms [65].

Despite these potential benefits, the available literature seems to indicate that the evidence for the supplementation of prebiotics for the prevention of allergies is not strong enough to make any clear recommendations yet, according to D’Auria et al. [66]. Finally, the use of prebiotics in cow milk allergy (CMA) appears to be a promising field of study according to Zepeda-Ortega et al. [67]. In this sense, they define that a healthy lifestyle and food diversity from pre-pregnancy, ensuring the adequate intake of dietary fiber, can aid in the primary prevention of CMA, especially in mothers with a history of allergic diseases and a planned c-section delivery. Similarly, in the clinical management of this condition, the use of extensive hydrolysate formulae with pre- and probiotics, together with long-chain polyunsaturated fatty acids, can aid and support early oral tolerance induction, although the use of this supplement for the primary prevention of CMA remains to be studied.

### 5.5. Skin Maladies

Beyond the digestive and respiratory tract, prebiotics may have a clinical use in preventing skin affections such as atopic dermatitis and skin allergy [68]. Skin maladies are multifactorial disorders related to different environmental factors such as diet, air pollution, sun exposure, activity level, etc., with nutritional interventions being one of the strongest factors that can be controlled to improve quality of life [69]. Evidence suggests that synbiotics consumption during pregnancy and lactation can decrease eczema incidence in offspring [70]. There have been few clinical trials with novel formulas evaluating eczema severity and manifestations in unweaned babies [35]. Although synbiotics are showing promising results related to atopic dermatitis, further studies regarding adequate dosing and formulas specific to colonic dysbiosis are still required [71].

A systematic review and meta-analysis evaluated 10 intervention trials with 4242 participants consuming prebiotic supplements either alone or in combination with other prebiotics or probiotics, and nine comparing infant formula milk with and without prebiotics, showing no clear evidence that prebiotic supplementation reduces eczema at age ≤ 4 years [72]. Furthermore, they reported that maternal, rather than infant, supplementation may be a more appropriate approach.

### 5.6. Protection against SARS-CoV-2

Scientific progress around the SARS-CoV-2 pandemic is the order of the day. The use of immunobiotics represent an interesting approach that is currently being explored to limit the dangers from SARS-CoV-2 infection (i.e., AM3, -NCT04987554-) Protective therapy with immunobiotics has also been discussed with regard to the potential application for the reinforcement of the microbiome in the fetus as part of the hypothesis that intrauterine programming is a key point for microbiome development [73].

Clinical data collected related to the use of prebiotics is summarized in Table 1.

## 6. Conclusions, Limitations, and Reflections

**Figure 3:**
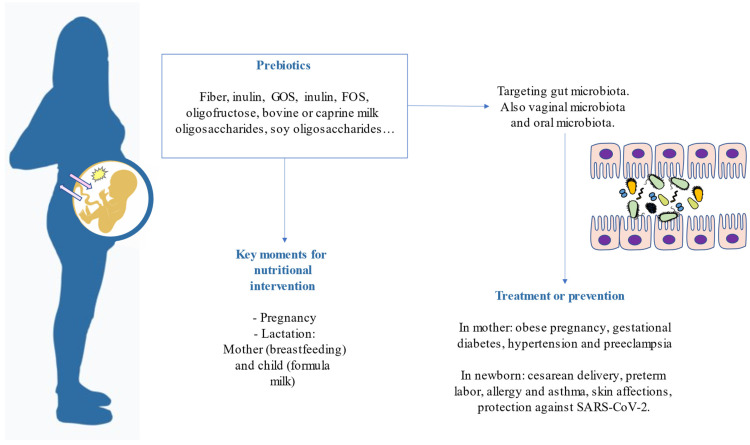
Highlights reviewed related to prebiotics in pregnancy and after birth.

From the route we have addressed throughout the manuscript, we can draw some conclusions. First, the nutritional status of the pregnant mother is key for the earliest microbial colonization in newborns. Therefore, betting on performing nutritional programs in pregnancy is taking care of both the mother and the baby. Secondly, prebiotics are feasible and cost-effective elements that can be added to a newborn’s diet through some milk or breast milk from supplemented mothers. Some clinical evidence alleges that these bioactive compounds may prevent allergies, colic, and other maladies in newborns. The combination of both prebiotics and probiotics is necessary to guarantee a proper infant gut microbiota colonization. Furthermore, in the mother, probiotic colonization is prosperous when combined with prebiotics and other micronutrients. These highlights are summarized in Figure 3.

Limitations in this narrative review include, firstly, the paucity of data available from articles in PubMed. Secondly, the search criteria included articles concerning pregnancy, lactation and newborn health. The objective of the present review has been to synthesize the information when searching about the effects of prebiotics in pregnancy and the postnatal period, in addition to considering the quality of lactation and the newborn’s health, starting from the prenatal period.

Conversely, the information provided here revolves around the favorable effect of prebiotics, treating them in isolation once the search criteria only included the word “prebiotics”. However, we must consider that in the scientific literature there is also therapeutic evidence with regard to eating patterns and dietary interventions based on the Mediterranean diet, which, as we know, contains a vast number of fruits and vegetables, which are foods rich in prebiotics. This is vitally important, as expert opinion alleges that nutrients and bioactive compounds have higher bioavailability in a food matrix that, when combined, may act synergistically. Therefore, in healthy women the preference should be healthy dietary patterns, and supplementation with prebiotic formulas should be considered when some requirements can only be met through the diet.

Furthermore, as these benefits are observable in the long term, scientists should also be encouraged to conduct long-term studies beginning with the newborn to adolescence or even young adulthood. Although there is no doubt regarding its safety, another issue in the inconsistent literature is that every individual presents a specific microbial composition, and that pro- and prebiotic supplementations should be specifically targeted to each gut.

When searching for scientific evidence regarding nutritional interventions in pregnant women, it seems that the current trend is to focus on supplementation with vitamins and minerals, but there are fewer hypotheses focusing on how to guarantee the establishment of a healthy microbiota from the prenatal period. In this sense, less is known about prebiotics; however, as with any nutrient, they do not have a relevant effect alone, but rather are essential components in a healthy dietary context. Moreover, they are fundamental pieces for probiotic colonization to develop a competent gut immune system and may contribute to the prevention of many maladies in the mother and child, in both prenatal and postnatal times.

The future of clinical management involves the application of integrative therapies that include a nutritionist for adjuvant treatment, especially in critical periods like pregnancy. Addressing different factors, including nutritional intervention, that concern the state of well-being of an individual, can assure better outcomes for their quality of life and reduce the risk of certain diseases. Higher awareness about lifestyle factors in the general population is forcing health systems to adopt measurements that include these kinds of strategies involving bioactive components such as the prebiotics discussed in this paper.

## 7. Methods of Searching

**Figure 4:**
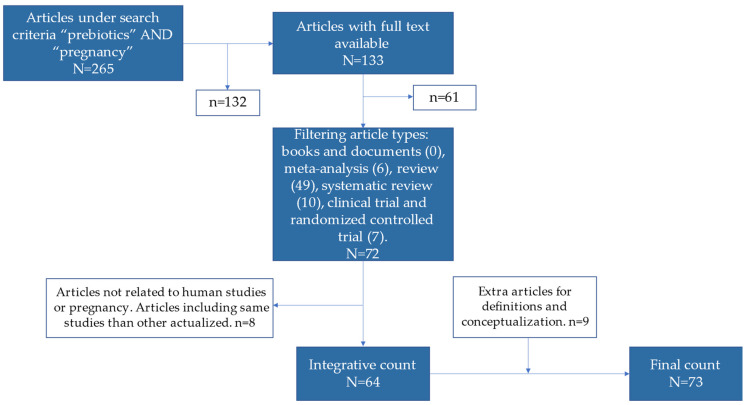
Flowchart of articles included in the narrative review. The picture follows the instructions from an integrative review based on PRISMA rules, but in the last step there are few articles added that convey a narrative review.

This was a narrative review explaining the current theoretical and experimental content available in the scientific literature relevant to the use of prebiotics in pregnancy. The *Pubmed* database was used exclusively, and the search criteria was mainly “prebiotics” [AND] “pregnancy”. A total of 265 publications were found under these criteria. The explanation related to clinical evidence followed the strategy of an integrative review applying the following filters in *Pubmed*: full text availability and article types including books and documents, meta-analysis, review, systematic reviews, clinical trials, and randomized controlled trials. Books and documents were not found; seven (randomized) clinical trials were found, in addition to 10 systematic reviews, six meta-analyses, and 49 reviews (that may include systematic, narrative and others). A total of 72 articles were screened, and then five articles were excluded for not being related to pregnancy, although they appeared in the search criteria due to matches with MeSH (Medical Subject Headings) terms in *Pubmed*. One was excluded for being an article on animal production, and one had “prebiotics” in the keywords but did not mention them in the text. One systematic review published in 2012 was discarded due to containing empirical articles from the previous decade, showing less consistency. The integrative count of articles included was 64. The oldest articles included in the search narrowing were from 2013, so the main content included concerns the last 10 years. There is an exceptional article from 1995 that explains the definition of “prebiotics”, and some extra articles (9) were added to the Introduction and other sections that deserved conceptualizations, constructing a narrative review instead of a merely integrative one. The final count of articles was 73. The selection of articles are schematized in Figure 4. The objective of the present review was to contrast the evidence and draw some guidelines that can be useful for the clinical management of pregnant woman in health and disease. The information has been classified depending on the topic of each article.

## Figures and Tables

**Table 1:** Evidence from clinical use of prebiotics in pregnancy, its complications, lactation and pediatric disorders.

	Condition	Evidence from Clinical Trials	References
**Pregnancy and its complications**	Healthy pregnancy	In maternal intake of FOS during the third trimester, there was a significant increase in fecal *Bifidobacterium* spp. and *Bifidobacterium longum* in the intervention group at the end of pregnancy. Administration of milk fortified with prebiotic, probiotic, DHA and micronutrients, observing notable increases in fecal concentration of the organism used as probiotic, alleging that probiotic colonization is successful when combined with prebiotics and other micronutrients. Comparing pregnant women for 9 weeks, synbiotics containing *Lactobacillus sporogenes* and inulin showed significant differences with lower serum insulin levels in the intervention group versus the control group. Regarding safety of pro- and prebiotics use in pregnancy and lactation, although only in some cases were changes in stool consistency noticed, but did not have serious effects for the mother or the infant’s health.	[18,19,20,21]
	Gestational diabetes (GDM)	Intervention with synbiotics has shown significant improvements on glucose and lipid metabolism, insulin resistance, as well as anti-inflammatory and antioxidant ability in diet controlled GDM patients, reducing the risk of fetal hyperbilirubinemia, fetal macrosomia, and limiting newborn weight. However, to optimize the results, more characterization about combinations with certain probiotics strains should be considered.	[29,30,31]
	Hypertension and preeclampsia	The safety of pro- and prebiotic use stimulates gut-derived metabolites, such as butyrate, that attenuate inflammation. There were no notable benefits from the administration of probiotics or synbiotics in women with hypertensive disorders or GDM, although due to the limited number of studies available, additional efforts are required.	[32,33]
	Bacterial vaginosis	Synbiotics consumption seems to effectively prevent recurrent urinary tract infections in women. In the case of bacterial vaginosis, they suggest that a combo of probiotics and prebiotics should be applied instead of using antibiotics, which is risky for a pregnant woman. Furthermore, pre/probiotic regimens seem to have even higher cure rates than antibiotics.	[35,36]
	Perinatal mental health	Limited evidence of a lower incidence of anxiety and depressive symptoms in the perinatal period has been reported when supplementing with pro-, pre- and synbiotics during pregnancy.	[37,38]
**Fortified formula milk and breastfeeding**)	Prevention in the window of opportunity	There are significant increases in fecal *Bifidobacterium* spp. when treating mothers with FOS, but no difference was observed in neonates aged 1 month. Longer term studies need to be undertaken. Short-term studies have observed reduced daily crying time when administering probiotics compared to placebo. The same studies defend the application of oligosaccharide prebiotics to promote the growth of beneficial bacteria as treatments for allergy or intolerance and for crying in babies with colic that are on formula. A premise from ecological theory says that microbial community development is affected by priority effects that determine species arrival and their behavior, which can be favorably modulated by pro- and prebiotics, so therapies targeting the gut microbiota are a potential choice. Different formulas of milk have reported an increase in bifidogenic bacteria and a decrease in opportunistic pathogens, in addition to a reduction of fecal pH, an increase in alpha diversity, and optimized calcium absorption. Human milk composition in prebiotics stimulate Bifidobacteria and Lactobacilli as well. However, when breast milk is not available or adequate, HMO fortified formulas seem to be the best option. A randomized controlled trial studied the effect of a specific prebiotic mixture administered from the day of birth on bifidobacterial and lactobacilli counts. An examination was performed at 3, 6 and 12 months of age, finding that the supplemented group had more fecal bifidobacterial and lactobacilli compared to placebo, these differences being maintained six months later without further supplementation. This kind of explorative study shows that prebiotics, even in the first days of life, are effective in establishing a competent gut immune system.	[18,43,44,46,47,48]
**Pediatric disorders**	Caesarean delivery	Stool samples from supplemented babies are softer and seem to be related to a lower level of pathogens. Nevertheless, the duration of supplementation to ensure a lasting beneficial effect is yet unknown, and another gap to close in clinical studies. A recent systematic review noted that the sooner the intervention during pregnancy and lactation after cesarean delivery, the better the effects that are achieved, especially *Bifidobacterium* colonization. Results were even more favorable in breastfed infants from supplemented mothers. The utilized formulas included GOS, FOS, or bovine milk-derived oligosaccharides, combined with probiotics from the genera *Lactobacillus*, *Bifidobacterium*, *Propionibacterium*, and *Streptococcus*.	[56,59]
	Asthma and allergy	The use of a formula milk containing a mixture of prebiotic oligosaccharides was associated with the lower incidence for recurrent wheezing in 132 infants at risk of atopy, although supplementation of non-human neutral and acidic oligosaccharides during the neonatal period did not reduce the incidence of allergies, bronchial hyper-reactivity, or respiratory infections in 113 preterm infants. They found a meta-analysis of two studies with 249 infants, reporting a reduction in infant asthma or recurrent wheezing in prebiotic-treated infants, and a single study reporting a significant reduction of the risk of developing food allergies by the use of prebiotics. These results are due to their ability to induce the production of short-chain fatty acids, especially dietary fiber and oligosaccharides, leading to the activation of T regulatory cells and tolerance mechanisms. Despite these potential benefits, the available literature seems to indicate that the evidence for the supplementation of prebiotics for the prevention of allergies are not yet strong enough to make any clear recommendations.	[64,65,66]
	Skin maladies	Synbiotics consumption during pregnancy and lactation seems to decrease eczema incidence in offspring. Few clinical trials have noticed lower eczema and atopic dermatitis severity in unweaned babies. Studies related to maternal supplement intake while lactating compared to using prebiotic-enriched formula milk, seem to be a more proper approach to reduce eczema in ≤4 year-aged babies.	[70,71,72]

## Data Availability

The data used to support the findings of the present study are available from the corresponding author upon request.
